# Identification of Anticoagulation Benefit Subgroups of Patients With Left Ventricular Thrombus

**DOI:** 10.31083/RCM27179

**Published:** 2025-06-26

**Authors:** Zechen Liu, Boqun Shi, Rui Zhang, Kefei Dou, Weihua Song

**Affiliations:** ^1^Fuwai Hospital, Chinese Academy of Medical Sciences & Peking Union Medical College/National Center for Cardiovascular Diseases, 100037 Beijing, China; ^2^Coronary Heart Disease Center, Department of Cardiology, Fuwai Hospital, Chinese Academy of Medical Sciences & Peking Union Medical College/National Center for Cardiovascular Diseases, 100037 Beijing, China; ^3^Cardiometabolic Medicine Center, Department of Cardiology, Fuwai Hospital, Chinese Academy of Medical Sciences & Peking Union Medical College/National Center for Cardiovascular Diseases, 100037 Beijing, China; ^4^Department of Cardiology, Beijing Anzhen Hospital, Capital Medical University, 100029 Beijing, China

**Keywords:** anticoagulation, cluster, left ventricular thrombus, left ventricular function

## Abstract

**Background:**

Left ventricular thrombus (LVT) is associated with major adverse cardiovascular and cerebrovascular events (MACCEs). Anticoagulation represents the current primary management for LVT; however, current studies in some Asian populations suggest that the anticoagulation benefit in LVT patients is not significant. Given the heterogeneity of clinical phenotypes in LVT patients, the population of LVT patients who benefit from anticoagulation needs to be further explored.

**Methods:**

This study included patients diagnosed with LVT at the FuWai Hospital from 2009 to 2021. We performed a latent class analysis (LCA) based on important clinical characteristics to objectively determine the number and dimensionality of clusters. Additionally, Kaplan–Meier curves and a Cox analysis were used to explore the relationship between anticoagulation therapy and MACCEs and major bleeding events in LVT patients.

**Results:**

A total of 1085 patients were enrolled in this study, and during a median follow-up time of 36.5 months, 206 patients developed MACCEs, while 16 patients developed major bleeding events. Moreover, 1085 patients were categorized into four clusters following the LCA. In the adjusted model, the risk of MACCEs was significantly lower in LVT patients receiving anticoagulation in cluster 4 (hazard ratio (HR): 0.486, 95% confidence interval (CI): 0.243–0.971) than in the group not receiving anticoagulation; however, there were no differences in the other three clusters or the whole population. There was a significant interaction between anticoagulation and the clustered subgroups (*p* for interaction in MACCEs: 0.046). However, no significant correlation was found for major bleeding events across clusters or for anticoagulant therapy.

**Conclusions:**

Our study suggests that not all LVT patients benefit from anticoagulation therapy; younger LVT patients with fewer complications and more cardiomyopathies are more likely to benefit from anticoagulation therapy.

## 1. Introduction

Left ventricular thrombus (LVT) is a severe complication associated with left ventricular dysfunction, which can arise from either ischemic or nonischemic cardiomyopathy. Its incidence was as high as 57% before the percutaneous coronary intervention (PCI) era [1, 2]. The incidence of LVT after myocardial infarction has been significantly reduced due to the widespread use of PCI, reperfusion, drugs that delay ventricular remodeling, and potent anticoagulation therapy over the last decades [3, 4]. However, despite these therapeutic advances, there is still a poor prognosis associated with LVT, which has been shown to be highly correlated with major adverse cardiovascular and cerebrovascular events (MACCEs) [5, 6, 7].

Anticoagulation is now widely used in the management LVT and has been shown in studies to improve the patient’s prognosis and reduce the incidence of MACCE [8, 9, 10]. However, in a study of the duration of anticoagulation in an Asian population, Goh *et al*. [11] found that prolonged anticoagulation in 412 patients with LVT failed to reduce the incidence of stroke. In their post hoc analysis, they determined that this may have been due to the inclusion of patients with a lower risk of baseline embolism [11, 12]. In another earlier study involving 648 patients with left ventricular aneurysms after a myocardial infarction (13.7% of them with LVT) there was no benefit of anticoagulation in patients with LVT [13]. The heterogeneity of anticoagulation therapy was also discussed in the AHA’s expert consensus [10]. Given the inconsistency of previous studies and the need for individualized treatment regimens, it is now believed that LVT patients are heterogeneous and that the benefit from anticoagulation varies from patient to patient, and that there is a gap in current research on this topic.

A study also suggests that there are different phenotypes associated with prognosis in LVT patients [14]. Therefore, based on the largest known LVT cohort in an Asian population, this study explores the subgroups of LVT patients more likely to benefit from anticoagulation therapy by using an artificial intelligence clustering method [15, 16].

## 2. Methods

### 2.1 Study Design, Population, and Data

**Fig. 1.**
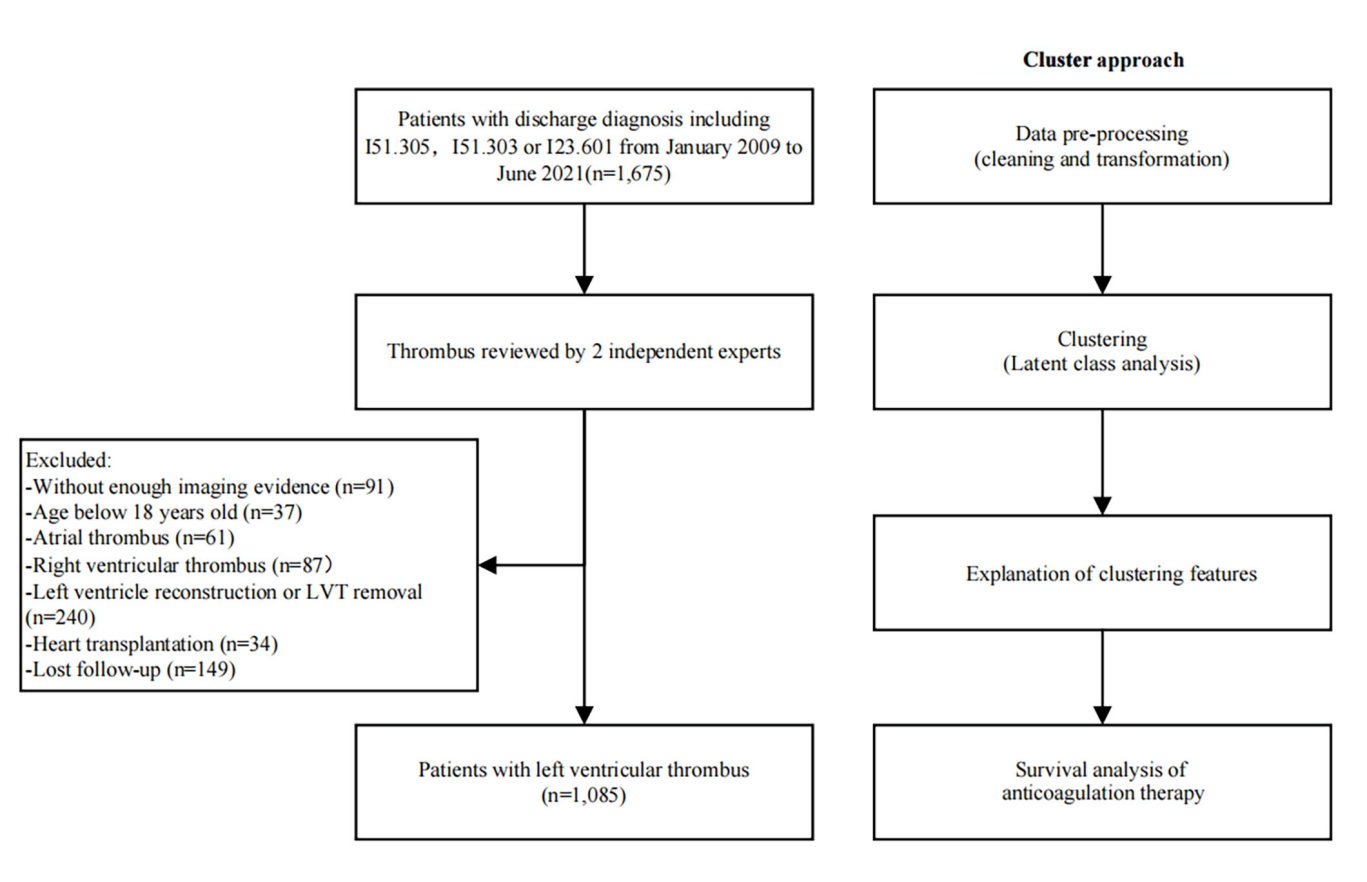
**Study flow chart**.

This study was retrospectively performed in the Fuwai Hospital, China, using individual patient data from patients hospitalized between January 2009 and June 2021. The Institutional Review Board at the Fuwai Hospital approved the use of clinical data for this study and waived the individual informed consent. Patients were eligible for inclusion by searching for the following terms and its ICD-10 (International Classification of Diseases 10th revision) code edition for clinical use in Beijing, in the discharge diagnosis in the electronic medical records system. These terms included: “mural thrombosis of the left ventricle (I51.305)”, “ventricular thrombus (I51.303)”, and “mural thrombosis of the left ventricle after acute myocardial infarction (I23.601)”. Patients who met any of the following criteria were excluded: (1) patients without enough imaging evidence; (2) patients who are less than 18 years old; (3) patients with atrial or right ventricular thrombus; (4) patients undergoing heart transplant, left ventricle reconstruction, or LVT removal during their hospital stay; (5) patients with incomplete follow-up data. The subject enrollment and analysis approach are illustrated in Fig. 1. Thrombus evaluations were depicted in the **Supplemental Methods**.

In addition, we noted other medications affecting cardiovascular prognosis, such as diabetes medications (including insulin, metformin, acarbose, dipeptidyl peptidase-4 inhibitors, glucagon-like peptide-1 analogs, and sodium-dependent glucose transporter-2 inhibitors), statins, angiotensin-converting enzyme inhibitors, β-blockers, and calcium channel blockers. To avoid the influence of treatment-related covariates, the use of the above 5 medications was collected on the basis of data from patients discharged from the hospital with medications.

### 2.2 Follow-up and Outcomes

The follow-up approach in this study was adapted from previous follow-up methods used in LVT studies to fit the characteristics of our cohort population [17]. We primarily determined whether the endpoint event had occurred through individual telephone contact conducted in the first quarter of 2022. For patients who could not be reached by phone, we extracted information from available medical records and reviewed it during their most recent outpatient visit. The interquartile range of the follow-up period was 1.9 to 6.6 years, with an overall follow-up rate of 91.1%. The outcome of this analysis was all-cause mortality, cardiovascular death, ischemic stroke, and MACCE. MACCE was the primary outcome, defined as the composite of cardiovascular death, ischemic stroke, and acute myocardial infarction (AMI) [18]. The major bleeding event was defined as a Bleeding Academic Research Consortium (BARC) type 2, 3, or 5 bleeding based on previous research and standardized bleeding definitions for cardiovascular clinical trials [19].

### 2.3 Latent Class Analysis (LCA) for Clustering

LCA has been employed to identify clusters of clinical characteristics. This statistical method is designed to elucidate the relationships between epistatic categorical variables by utilizing a minimal number of latent classes while ensuring local independence among the epistatic variables within each class [16, 20, 21]. LCA is frequently applied to identify distinct subgroups within a population, allowing for the characterization of these subgroups and their association with clinical outcomes. The selection of clinical variables for LCA was determined a priori by integrating categorical variables that have been linked to prognosis in patients with LVT in standard clinical practice. These variables include antiplatelet therapy, hypertension, dyslipidemia, diabetes mellitus, CKD history, prior myocardial infarction (MI), LVEF ≤40%, apical LVT, round LVT, mobile LVT, multiple LVT, cardiomyopathy, estimated glomerular filtration rate (eGFR) ≥60 mL/min, body mass index ≥24, age ≥65, and gender. The clustering of all patients was performed through 10 iterations of maximum likelihood estimation. To ascertain the optimal number of subgroups, ranging from 2 to 7, we utilized the akaike information criterion (AIC), the bayesian information criterion (BIC), and the sample was size-adjusted. We determine the final number of clustering groups after ensuring that the number of clusters in each cluster is not less than 5% of the total and combining the optimal AIC and BIC. These criteria facilitate the identification of the most parsimonious model that adequately represents the data, balancing model fit with complexity.

### 2.4 Statistical Analysis

Summary results are presented as percentages or median. Categorical variables were compared using the χ^2^ test or Fisher’s exact test, and continuous variables were compared using the Mann-Whitney test. Kaplan-Meier (KM) survival analysis and the log-rank test were used to compare the clusters or therapy across various endpoint definitions. The results of univariate and multivariate Cox regression proportional risk analyses were presented as 95% confidence intervals (CI) and hazard ratios (HR). A two-tailed *p* value of 0.05 was considered statistically significant. All data preparation and analyses used R version 4.3.0 (R Foundation for Statistical Computing, Vienna, Austria). The R packages needed for the statistical processes are shown in** Supplemental Methods**.

## 3. Results

Data abstraction was completed for 1675 patients and baseline data grouped by anticoagulation therapy are presented in **Supplementary Table 1**. The overall follow-up rate was 91.1%. Finally, 1085 patients with LVT were included, with an average age of 53.7 years and LVEF of 37.6%. 905 (83.4%) of patients were male. With a median follow-up time of 36.5 months, a total of 206 patients developed MACCE, including 167 cardiovascular deaths, 32 strokes, and 18 AMIs, with 2 patients combining stroke and AMI culminating in cardiovascular death, and 7 patients with cardiovascular death after AMI. In addition, major bleeding events occurred in 16 patients.

### 3.1 Identification of Four Clusters

In determining the optimal number of clusters for clusters 2–7, we selected the smallest AIC and BIC values, ultimately deciding on a four-cluster solution. The specific number of clusters and their corresponding AIC and BIC values are detailed in **Supplementary Table 2**. Of the 1085 patients analyzed, 57 (5.3%) were categorized in cluster 1, 147 (13.5%) in cluster 2, 652 (60.1%) in cluster 3, and 229 (21.1%) in cluster 4.

There were notable differences in the baseline characteristics among the four patient clusters, with the key distinctions outlined below and the detailed data presented in Table 1. Patients in cluster 4 were significantly younger and had a higher proportion of females compared to the other clusters. They also exhibited lower body mass index and systolic blood pressure levels, along with a higher heart rate. Regarding past medical history, cluster 4 had the lowest prevalence of hypertension, diabetes mellitus, peripheral artery disease, and prior stroke. Additionally, this cluster had the lowest rates of prior MI, prior coronary artery bypass grafting (CABG), and prior PCI. In terms of presenting diseases, cluster 4 patients were more likely to have cardiomyopathy, including dilated, hypertrophic, and other forms, rather than coronary artery disease. Regarding medication, a higher number of patients in cluster 4 received antiplatelet therapy in comparison to anticoagulation therapy alone. Imaging differences were also pronounced. Collectively, patients in cluster 4 had a higher presence of hypokinesis and akinesis. They also had a smaller percentage of apical, round, mobile, and multiple LVT.

**Table 1.** **Baseline features in different clusters**.

		Total	Cluster 1	Cluster 2	Cluster 3	Cluster 4	*p* value
n (%)		1085	57 (5.3)	147 (13.5)	652 (60.1)	229 (21.1)	
Demographic							
	Age (median [IQR]) (years)	55.0 [45.0, 64.0]	47.0 [41.0, 57.0]	68.0 [58.0, 74.0]	56.0 [48.0, 64.0]	43.0 [30.0, 53.0]	<0.001
	Male (%)	905 (83.4)	53 (93.0)	109 (74.1)	592 (90.8)	151 (65.9)	<0.001
	BMI (median [IQR]) (kg/m^2^)	25.1 [22.8, 27.5]	25.8 [24.2, 28.7]	25.0 [22.7, 27.2]	25.4 [23.2, 27.6]	23.7 [21.6, 27.3]	<0.001
	Heart rate (median [IQR]) (BPM)	76.0 [67.0, 87.0]	80.0 [75.0, 89.0]	75.0 [68.0, 87.0]	73.0 [64.0, 83.0]	82.0 [71.0, 96.0]	<0.001
	SBP (median [IQR]) (mmHg)	115.0 [103.0, 130.0]	119.0 [108.0, 129.0]	117.0 [106.5, 130.0]	120.0 [106.0, 130.3]	106.0 [97.0, 116.0]	<0.001
	DBP (median [IQR]) (mmHg)	74.0 [67.0, 82.0]	80.0 [70.0, 90.0]	70.0 [65.0, 80.0]	75.0 [68.0, 82.0]	71.0 [65.0, 81.0]	<0.001
Past medical history							
	Dyslipidemia (%)	752 (69.3)	50 (87.7)	128 (87.1)	551 (84.5)	23 (10.0)	<0.001
	Hypertension (%)	519 (47.8)	40 (70.2)	102 (69.4)	340 (52.1)	37 (16.2)	<0.001
	Diabetes mellitus (%)	441 (40.6)	36 (63.2)	91 (61.9)	249 (38.2)	65 (28.4)	<0.001
	eGFR <60 mL/min/1.73 m^2^ (%)	165 (15.2)	13 (22.8)	122 (83.0)	1 (0.2)	29 (12.7)	<0.001
	Peripheral artery disease (%)	81 (7.5)	7 (12.3)	16 (10.9)	49 (7.5)	9 (3.9)	0.036
	Prior stroke (%)	170 (15.7)	14 (24.6)	38 (25.9)	96 (14.7)	22 (9.6)	<0.001
	Prior MI (%)	583 (53.7)	7 (12.3)	114 (77.6)	453 (69.5)	9 (3.9)	<0.001
	Prior CABG (%)	23 (2.1)	1 (1.8)	10 (6.8)	12 (1.8)	0 (0.0)	<0.001
	Prior PCI (%)	170 (15.7)	3 (5.3)	39 (26.5)	128 (19.6)	0 (0.0)	<0.001
	Prior cerebral hemorrhage (%)	8 (0.7)	2 (3.5)	0 (0.0)	4 (0.6)	2 (0.9)	0.110
	Atrial fibrillation (%)	96 (8.8)	5 (8.8)	23 (15.6)	47 (7.2)	21 (9.2)	0.020
Underlying disease							
	Coronary artery disease (%)	830 (76.5)	24 (42.1)	142 (96.6)	637 (97.7)	27 (11.8)	<0.001
	STEMI (%)	235 (21.7)	2 (3.5)	36 (24.5)	192 (29.4)	5 (2.2)	<0.001
	NSTEMI (%)	46 (4.2)	1 (1.8)	19 (12.9)	24 (3.7)	2 (0.9)	<0.001
	Cardiomyopathy (%)	205 (18.9)	38 (66.7)	3 (2.0)	8 (1.2)	156 (68.1)	<0.001
	Dilated cardiomyopathy (%)	173 (15.9)	33 (57.9)	2 (1.4)	1 (0.2)	137 (59.8)	<0.001
	Hypertrophic cardiomyopathy (%)	26 (2.4)	2 (3.5)	1 (0.7)	5 (0.8)	18 (7.9)	<0.001
	Perinatal cardiomyopathy (%)	16 (1.5)	0 (0.0)	0 (0.0)	0 (0.0)	16 (7.0)	<0.001
	Restrictive cardiomyopathy (%)	6 (0.6)	0 (0.0)	1 (0.7)	0 (0.0)	5 (2.2)	0.003
	Alcoholic cardiomyopathy (%)	14 (1.3)	1 (1.8)	2 (1.4)	1 (0.2)	10 (4.4)	<0.001
	Myocarditis (%)	6 (0.6)	0 (0.0)	0 (0.0)	1 (0.2)	5 (2.2)	0.012
	NVM (%)	26 (2.4)	4 (7.0)	2 (1.4)	5 (0.8)	15 (6.6)	<0.001
Medications at discharge							
	Antiplatelet therapy (%)	725 (66.8)	8 (14.0)	121 (82.3)	579 (88.8)	17 (7.4)	<0.001
	Aspirin (%)	617 (56.9)	7 (12.3)	93 (63.3)	501 (76.8)	16 (7.0)	<0.001
	Clopidogrel (%)	512 (47.2)	2 (3.5)	90 (61.2)	417 (64.0)	3 (1.3)	<0.001
	Ticagrelor (%)	41 (3.8)	0 (0.0)	6 (4.1)	35 (5.4)	0 (0.0)	0.001
	DAPT (%)	445 (41.0)	1 (1.8)	68 (46.3)	374 (57.4)	2 (0.9)	<0.001
	Anticoagulation therapy (%)	639 (58.9)	50 (87.7)	83 (56.5)	310 (47.5)	196 (85.6)	<0.001
Anticoagulation status							
	Warfarin (%)	382 (35.2)	24 (42.1)	52 (35.4)	185 (28.4)	121 (52.8)	<0.001
	Apixaban (%)	1 (0.1)	0 (0.0)	0 (0.0)	0 (0.0)	1 (0.4)	0.399
	Dabigatran (%)	22 (2.0)	2 (3.5)	0	11 (1.7)	9 (3.9)	0.028
	Rivaroxaban (%)	234 (21.6)	24 (42.1)	31 (21.1)	114 (17.5)	65 (28.4)	<0.001
Other medicines use							
	DM medicine (%)	165 (15.2)	9 (15.8)	42 (28.6)	101 (15.5)	13 (5.7)	<0.001
	ACEI (%)	715 (65.9)	43 (75.4)	76 (51.7)	455 (69.8)	141 (61.6)	<0.001
	β-Blocker (%)	861 (79.4)	44 (77.2)	122 (83.0)	518 (79.4)	177 (77.3)	0.583
	CCB (%)	73 (6.7)	6 (10.5)	12 (8.2)	48 (7.4)	7 (3.1)	0.068
	Statins (%)	774 (71.3)	35 (61.4)	124 (84.4)	570 (87.4)	45 (19.7)	<0.001
Imageological examination							
	LVEDD (median [IQR])	58.0 [53.0, 66.0]	67.0 [60.0, 75.0]	58.0 [53.0, 63.0]	56.0 [51.0, 61.0]	68.0 [60.0, 74.7]	<0.001
	LVEF (median [IQR])	38.0 [29.0, 46.0]	26.0 [22.0, 30.0]	35.0 [30.0, 40.5]	42.0 [35.0, 49.0]	26.0 [22.0, 31.0]	<0.001
	LVEF ≤40% (%)	667 (61.5)	295 (77.4)	305 (50.0)	43 (74.1)	11 (57.9)	<0.001
	Global hypokinesis (%)	279 (25.7)	43 (75.4)	21 (14.3)	48 (7.4)	167 (72.9)	<0.001
	Hypokinesis (%)	465 (42.9)	8 (14.0)	71 (48.3)	358 (54.9)	28 (12.2)	<0.001
	Akinesis (%)	652 (60.1)	13 (22.8)	118 (80.3)	492 (75.5)	29 (12.7)	<0.001
	Apical LVT (%)	986 (90.9)	54 (94.7)	133 (90.5)	604 (92.6)	195 (85.2)	0.006
	Round LVT (%)	656 (60.5)	57 (100.0)	76 (51.7)	373 (57.2)	150 (65.5)	<0.001
	Mobile LVT (%)	90 (8.3)	27 (47.4)	4 (2.7)	25 (3.8)	34 (14.8)	<0.001
	Multiple LVT (%)	119 (11.0)	36 (63.2)	7 (4.8)	23 (3.5)	53 (23.1)	<0.001
	LVT largest diameter (median [IQR]) (mm)	23.0 [17.0, 32.0]	22.0 [17.0, 31.0]	25.9 [18.0, 34.5]	23.0 [16.0, 31.0]	24.0 [16.0, 30.0]	0.092
	LVT area (median [IQR]) (mm^2^)	3.0 [1.7, 4.8]	3.4 [1.7, 6.2]	3.2 [1.8, 5.5]	2.9 [1.6, 4.5]	3.3 [1.7, 4.8]	0.099

Abbreviations: ACEI, angiotensin-converting enzyme inhibitor; BPM, beats per minute; BMI, body mass index; CCB, calcium channel blocker; DBP, diastolic blood pressure; DM, diabetes mellitus; eGFR, estimated glomerular filtration rate; MI, myocardial infarction; CABG, coronary artery bypass grafting; PCI, percutaneous coronary intervention; SBP, systolic blood pressure; STEMI, ST-segment elevation myocardial infarction; NSTEMI, non-ST-segment elevation myocardial infarction; NVM, noncompaction of the ventricular myocardium; DAPT, dual antiplatelet therapy; LVT, left ventricular thrombus; LVEDD, left ventricular end diastolic dimension; LVEF, left ventricular ejection fraction; IQR, interquartile range.

**Fig. 2.**
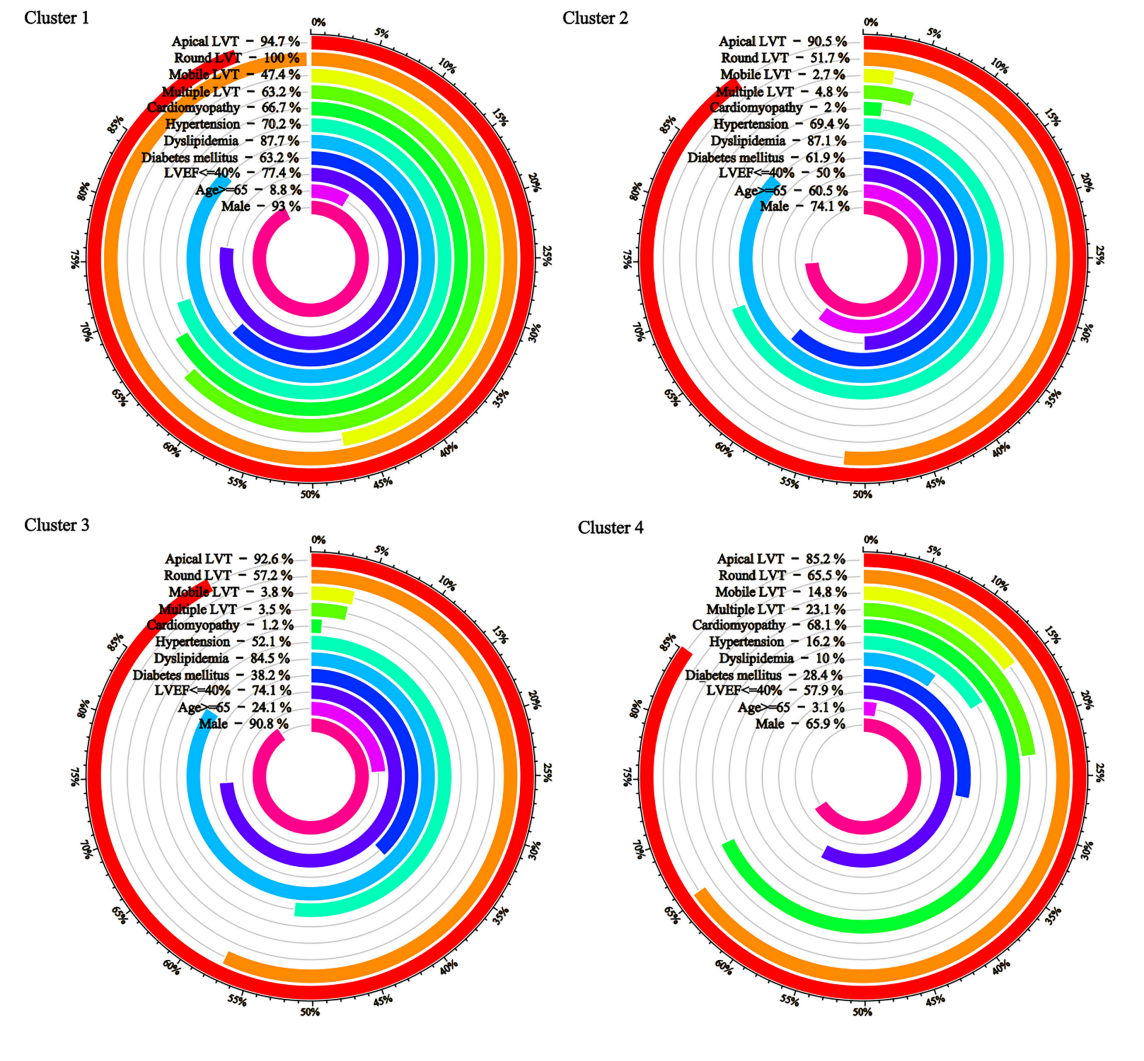
**Key baseline characteristics by cluster**.

Fig. 2 plots the bars according to the main dichotomous clustering features. Cluster 1 is the high comorbidity with more special forms of thrombus, cluster 2 is the older, more comorbidity, less special forms of thrombus, cluster 3 is the less comorbidity, less special forms of thrombus, and cluster 4 is the younger, female, less comorbidity, and more cardiomyopathy.

### 3.2 Association With Outcomes

**Fig. 3.**
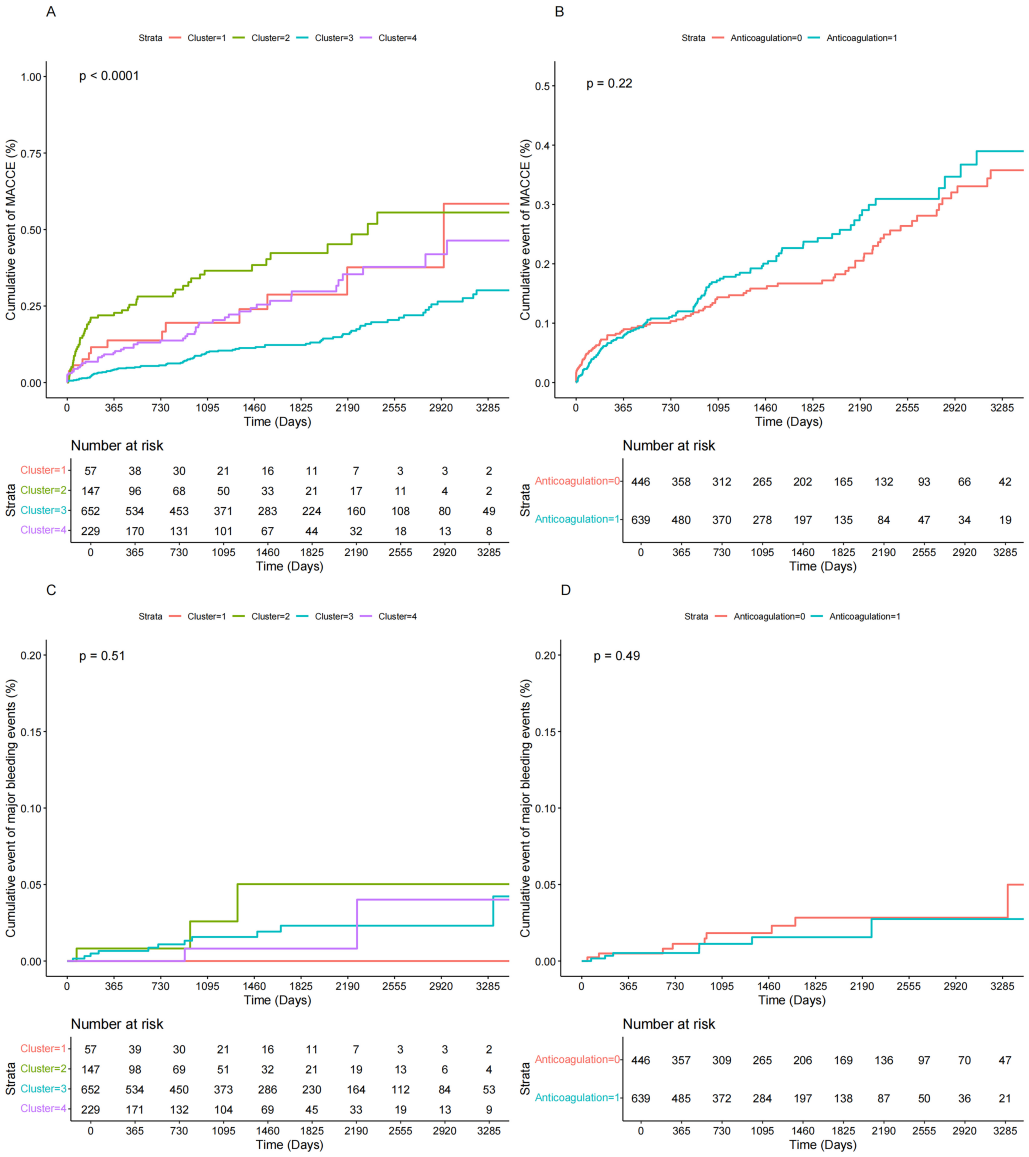
**Kaplan-Meier plots the risk of MACCE and major bleeding events in cluster and anticoagulation groups**. (A) Kaplan-Meier plots for the risk of MACCE in clusters. (B) Kaplan-Meier plots for the risk of MACCE in anticoagulation groups. (C) Kaplan-Meier plots for the risk of major bleeding events in clusters. (D) Kaplan-Meier plots for the risk of major bleeding events in anticoagulation groups.

Fig. 3 demonstrates the clinical outcome survival curves according to anticoagulation therapy or cluster. No significant event differences were found between the anticoagulation and non-anticoagulation groups for either MACCE or major bleeding events in the whole population (log-rank *p* for MACCE: 0.22; log-rank *p* for major bleeding events: 0.49). In the cluster, as to the cumulative event of MACCE, the KM curve of cluster 3 was steadily lower than that of the other 3 clusters during the follow-up period, whereas the event rate of cluster 1 eventually exceeded that of cluster 2 with the prolongation of the follow-up period (log-rank *p* for MACCE: <0.001). Possibly due to the low number of major bleeding events, no significant differences between clusters were found in the KM curves (log-rank *p* for major bleeding events: 0.51).

The outcomes of the Cox analysis and the corresponding forest plot are depicted in Table 2. Among the entire cohort of patients with LVT, anticoagulation therapy did not significantly mitigate the risk of MACCE or major bleeding events compared to those who did not receive anticoagulation. There was no significant decrease in MACCE in cluster 1 (HR: 0.849, 95% CI: 0.182–3.967, *p* = 0.835), cluster 2 (HR: 0.614, 95% CI: 0.353–1.068, *p* = 0.084), and cluster 3 (HR: 1.449, 95% CI: 0.942–2.228, *p* = 0.091) in model 1. However, in cluster 4, there was a statistically significant reduction in MACCE in anticoagulation therapy (HR: 0.489, 95% CI: 0.250–0.956, *p* = 0.036). In model 2, we further adjusted for the use of diabetes medications, statins, angiotensin-converting enzyme inhibitors, β-blockers, and calcium channel blockers, which may affect cardiovascular prognosis, and still observed a significant reduction in MACCE with anticoagulation in cluster 4 (HR: 0.486, 95% CI: 0.243–0.971, *p* = 0.041). Besides, in both the adjusted and unadjusted models, there was an interaction between anticoagulation therapy and cluster (*p* for interaction in model 1: 0.012; *p* for interaction in model 2: 0.046).

**Table 2.** **Cox analysis for MACCE among 4 clusters**.

		Model 1			Model 2		
		HR (95% CI)	*p* value	*p* for interaction	HR (95% CI)	*p* value	*p* for interaction
Cluster				0.012			0.046
	Cluster 1	0.849 (0.182, 3.967)	0.835		3.592 (0.557, 23.169)	0.179	
	Cluster 2	0.614 (0.353, 1.068)	0.084		0.764 (0.418, 1.398)	0.383	
	Cluster 3	1.449 (0.942, 2.228)	0.091		1.472 (0.957, 2.264)	0.078	
	Cluster 4	0.489 (0.250, 0.956)	0.036		0.486 (0.243, 0.971)	0.041	

Abbreviations: CI, confidence intervals; HR, hazard ratios; MACCE, major adverse cardiovascular and cerebrovascular event.

## 4. Discussion

Based on the largest known LVT cohort, this study incorporates the LCA approach to cluster analysis based on clinical and imaging phenotypes in the enrolled population and explores the clinical benefit of anticoagulation between clusters. The study showed that we found a cluster in which receiving anticoagulation significantly reduced MACCE with an interaction effect in the absence of a significant anticoagulation benefit in the population as a whole. In addition, no association between anticoagulation and major bleeding events was observed in the whole population or in individual clusters.

Previous studies have suggested that anticoagulation reduces the occurrence of MACCE in patients with LVT; however, no significant benefit of anticoagulation on clinical outcomes was observed in several cohort studies of Asian LVT patients, and the results of the analyses may be related to a lower risk of embolism at baseline and a lower overall rate of embolic events [11, 12]. This is consistent with the present study, in which we also did not observe a significant reduction in MACCE in patients receiving anticoagulation. However, anticoagulation is required based on pathophysiologic mechanisms, and therefore determining which patients are more likely to benefit from anticoagulation in the clinical setting is a question that needs to be addressed [6, 10]. A recent study, based on unsupervised machine learning, explored different clinical phenotypes in patients with LVT and suggested that they may be associated with different prognoses, but the study lacked follow-up data and did not explore the optimal population that benefited from anticoagulation [14].

In this study, the population that achieved statistically significant benefit from anticoagulation was the patients in cluster 4. The main characteristics were that patients in cluster 4 were younger, more likely to be female, and had less diabetes, hypertension, hyperlipidemia, and more cardiomyopathy (most notably dilated cardiomyopathy (DCM)) compared with the other clusters. The data from previous studies and our own results have allowed us to make several observations and interpretations on LVT patients receiving anticoagulation therapy. LVT patients with fewer comorbidities are more likely to benefit from anticoagulation. In addition, patients with DCM may also have potential benefits from anticoagulation. Indeed, this population corresponds to the clinically reported DCM patients with low comorbidities diagnosed with LVT [22]. This population, because of its low comorbidity, has a relative lack of Virchow’s triad that leads to thrombosis compared with the other groups, so adverse effects due to LVT are more easily corrected by anticoagulant therapy [23]. Unlike ischemic heart disease, the proportion of patients with DCM with LVT did not significantly decrease with improved treatment modalities [5, 24]. Combining retrospective data and prospective observational studies, Levine GN *et al.* [10] concluded that anticoagulation in patients with DCM combined with LVT should be continued for at least 3 to 6 months and needs to be adjusted for LVEF and adverse bleeding events.

In the survival analysis, we noted that the population with the worst prognosis following longer follow-up was the patients in cluster 1. These patients had the most cardiovascular comorbidities, the poorest left ventricular function, and the most specific types of thrombus. Experimental and clinical studies have demonstrated a link between heart failure and hypercoagulable states, with an increased incidence of stroke and other thromboembolic events in patients with heart failure and more comorbidities were associated with a poor prognosis [25]. Therefore, we hypothesized that for the management of LVT patients with multiple comorbidities, anticoagulation would be less protective against the occurrence of MACCE in LVT patients. In contrast, the population with the best prognosis belonged to cluster 3. This population has less comorbidities and specific types of thrombus in all four clusters, while having the least percentage of cardiomyopathy. This suggests another perspective on the impact of the presence of cardiomyopathy on the benefit from anticoagulation in patients with LVT.

To illustrate the assessment of clinical characteristics associated with anticoagulation efficacy in a higher dimension, we designed and employed a new combination of machine learning methods in this study. Traditional subgroup analyses differ from clinical practice because each individual’s risk does not occur in a binary fashion and may be co-correlated or clustered with other concomitant risk factors. Therefore, patient traits cannot be judged by a single characteristic, and it is more consistent with clinical practice to combine the clinical and imaging characteristics of patients to assign individualized treatment plans. We carefully screened dichotomous variables that included demographic characteristics, medical history, and LVT imaging features. We prioritized clusters with the lowest AIC and BIC values and ensured that each cluster contained no less than 5% of the total sample size to safeguard the scientific validity and interpretability of the clusters. Finally, a larger sample size and more complete follow-up data will help to answer the clinical questions.

In order to search objectively for underlying clinical trends, we used an unsupervised technique with clustering of LCA in this study. Our research, however, has a few limitations. First, in the selection of variables for inclusion in the LCA analysis, due to methodological constraints, it was inevitable that some continuous variable impacts were overlooked. Although significant continuous variables were converted into categorical variables and incorporated into the analysis based on their relevance, it remains possible that variables with potential prognostic or clustering influence were inadvertently excluded. Second, additional factors, such as imaging changes during follow-up that were not taken into account in our models, might also aid in the improvement of the clusters. Third, patients in different clusters had different treatment regimens and medications, and although we included the use of antiplatelet medications that were most likely to affect prognosis in our LCA analysis, in addition to correcting for medication profiles for treatment of diabetes, hypertension, and heart failure that were also likely to affect prognosis in subsequent survival analyses, it was still difficult to completely eliminate the prognostic impact of treatment differences. Finally, we lacked a sizable independent cohort to test our clustering results. We cannot conclude that the new clustering corresponds to a different LVT pathophysiology or that this clustering represents the most accurate classification of LVT subtypes available. Therefore, prospective validation of the results and clinical implications of this study is needed.

## 5. Conclusions

To better identify treatment response clusters for anticoagulant therapy utilized in patients with LVT, this study has shown the potential clinical usefulness of combining LCA-based techniques. Patients with younger, fewer complications, worse left ventricular function, and more cardiomyopathies are more likely to benefit from anticoagulation therapy. The findings demand further confirmation across different therapies and medical conditions, followed by a prospective study to see whether employing these techniques to guide therapy can enhance patient outcomes.

## Availability of Data and Materials

The data presented in this study are available on request from the corresponding author. The data are not publicly available due to privacy and ethical restrictions.

## Supplementary Material

Supplementary material associated with this article can be found, in the online version, at https://doi.org/10.31083/RCM27179.
