# Supplementary material for: Identification of Anticoagulation Benefit Subgroups of Patients With Left Ventricular Thrombus
Source: Rev Cardiovasc Med. 2025 Jun 26;26(6):27179. doi: 10.31083/RCM27179 (PMC12230831; doi:10.31083/RCM27179)
Supplement: Supplementary file 1 [file 2153-8174-26-6-27179-s1.zip › Supplementary Material.docx]

**SUPPLEMENTAL MATERIALS**

**1. Supplemental Methods**

**1.1 Variables included in the analysis**

The variables included in the analysis are: age, gender, body mass index, hypertension, diabetes mellitus, eGFR<60ml/min/1.73m^2^, peripheral artery disease, prior stroke, prior MI, prior CABG, prior PCI, prior cerebral hemorrhage, atrial fibrillation, coronary artery disease, STEMI, NSTEMI, dilated cardiomyopathy, hypertrophic cardiomyopathy, ARVD with associated LV impairment, perinatal cardiomyopathy, restrictive cardiomyopathy, alcoholic cardiomyopathy, myocarditis, NVM, LVEDD, LVEF, LVEF<=40%, global hypokinesis, hypokinesis, akinesis, apical LVT, round LVT, mobile LVT, multiple LVT, calcified LVT, LVT largest diameter, LVT area, left ventricular aneurysm, heart rate, systolic blood pressure, diastolic blood pressure. The full name of the abbreviation is the same as in Table 1.

**1.2 Thrombus evaluation**

This study assessed LVT using transthoracic echocardiography (TTE), contrast-enhanced computed tomography (CT), or cardiac magnetic resonance imaging (CMR). All patients underwent TTE at least once during hospitalization. LVT shows as an echo-dense mass with borders different from the endocardium and is frequently adjacent to a segment contracting unnaturally. The attending physicians chose whether to perform CMR or contrast-enhanced CT. Two experienced cardiologists independently interpreted the imaging data. They determined the location, shape, activity, and whether the LVT was multiple or calcified. A round LVT was defined as a thrombus with a protruding element that is not entirely attached to the ventricle wall. Left ventricular characteristics were also collected, including left ventricular end-diastolic dimension (LVEDD), LVEF, and wall motion.

**1.3 R packages needed for statistical analysis**

"readxl", "writexl", "flexclust", "openxlsx", "tableone", "dplyr", "survival", "survminer", "VIM", "mice", "zoo", "adjustedCurves", "foreign", "survminer", "reportReg", "rms", "ggsci", "glmnet", "corrplot", "wesanderson", "tidyr", "ggplot2",and "poLCA".

**2. Supplemental Tables**

**Supplementary Table 1 Baseline characters of 1675 patients**

|  | Total | Non-Anticoagulation | Anticoagulation | P value |
| --- | --- | --- | --- | --- |
| n (%) | 1675 | 763 | 912 |  |
| Demographic |  |  |  |  |
| Age (median[IQR]) (years) | 54.0[44.0,63.0] | 57.0[47.0,65.0] | 51.5[41.0,61.0] | <0.001 |
| Male (%) | 1387(82.8) | 640(83.9) | 747(81.9) | 0.287 |
| BMI (median[IQR]) (Kg/m2) | 24.8[22.5,27.3] | 24.8[22.5,27.0] | 24.8[22.4,27.6] | 0.131 |
| Heart rate (median[IQR]) (BPM) | 78.0[68.0,86.0] | 76.0[67.0,82.0] | 78.0[68.0,89.0] | 0.001 |
| SBP (median[IQR]) (mmHg) | 115.0[103.0,127.0] | 115.9[104.0,127.5] | 114.0[102.0,126.0] | 0.055 |
| DBP (median[IQR]) (mmHg) | 74.0[67.0,80.0] | 74.0[67.0,80.0] | 74.2[66.0,81.0] | 0.266 |
| Past medical history |  |  |  |  |
| Dyslipidemia (%) | 988(59.0) | 466(61.1) | 522(57.2) | 0.112 |
| Hypertension (%) | 727(43.4) | 344(45.1) | 383(42.0) | 0.204 |
| Diabetes mellitus (%) | 666(39.8) | 304(39.8) | 362(39.7) | 0.950 |
| eGFR<60 ml/min/1.73m2 (%) | 262(15.6) | 119(15.6) | 143(15.7) | 0.963 |
| Peripheral artery disease (%) | 106(6.3) | 42(5.5) | 64(7.0) | 0.205 |
| Prior stroke (%) | 250(14.9) | 112(14.7) | 138(15.1) | 0.796 |
| Prior MI (%) | 912(54.4) | 491(64.4) | 421(46.2) | <0.001 |
| Prior CABG (%) | 26(1.6) | 14(1.8) | 12(1.3) | 0.392 |
| Prior PCI (%) | 243(14.5) | 120(15.7) | 123(13.5) | 0.195 |
| Prior cerebral hemorrhage (%) | 10(0.6) | 5(0.7) | 5(0.5) | 1.000 |
| Atrial fibrillation (%) | 153(9.1) | 44(5.8) | 109(12.0) | <0.001 |
| Underlying disease |  |  |  |  |
| Coronary artery disease (%) | 1226(73.2) | 655(85.8) | 571(62.6) | <0.001 |
| STEMI (%) | 283(16.9) | 167(21.9) | 116(12.7) | <0.001 |
| NSTEMI (%) | 54(3.2) | 26(3.4) | 28(3.1) | 0.697 |
| Cardiomyopathy (%) | 315(18.8) | 71(9.3) | 244(26.8) | <0.001 |
| Dilated cardiomyopathy (%) | 270(16.1) | 62(8.1) | 208(22.8) | <0.001 |
| Hypertrophic cardiomyopathy (%) | 35(2.1) | 5(0.7) | 30(3.3) | <0.001 |
| Perinatal cardiomyopathy (%) | 19(1.1) | 4(0.5) | 15(1.6) | 0.036 |
| Restrictive cardiomyopathy (%) | 9(0.5) | 1(0.1) | 8(0.9) | 0.045 |
| Alcoholic cardiomyopathy (%) | 18(1.1) | 2(0.3) | 16(1.8) | 0.003 |
| Myocarditis (%) | 22(1.3) | 9(1.2) | 13(1.4) | 0.660 |
| NVM (%) | 38(2.3) | 10(1.3) | 28(3.1) | 0.016 |
| Medications at discharge |  |  |  |  |
| Antiplatelet therapy (%) | 1070(63.9) | 626(82.0) | 444(48.7) | <0.001 |
| Aspirin (%) | 941(56.2) | 611(80.1) | 330(36.2) | <0.001 |
| Clopidogrel (%) | 663(39.6) | 401(52.6) | 262(28.7) | <0.001 |
| Ticagrelor (%) | 57(3.4) | 49(6.4) | 8(0.9) | <0.001 |
| DAPT (%) | 591(35.3) | 435(57.0) | 156(17.1) | <0.001 |
| Anticoagulation therapy (%) | 1675 | 763 | 912 | <0.001 |
| Anticoagulation status |  |  |  |  |
| Warfarin (%) | 555(33.1) | 0(0.0) | 555(60.9) | <0.001 |
| Apixaban (%) | 1(0.1) | 0(0.0) | 1(0.1) | 1.000 |
| Dabigatran (%) | 30(1.8) | 0(0.0) | 30(3.3) | <0.001 |
| Rivaroxaban (%) | 307(18.3) | 0(0.0) | 307(33.7) | <0.001 |
| Other medicines use |  |  |  |  |
| DM medicine (%) | 194(11.6) | 91(11.9) | 103(11.3) | 0.687 |
| ACEI (%) | 881(52.6) | 353(46.3) | 528(57.9) | <0.001 |
| β-Blocker (%) | 1156(69.0) | 470(61.6) | 686(75.2) | <0.001 |
| CCB (%) | 94(5.6) | 43(5.6) | 51(5.6) | 0.969 |
| Statins (%) | 1016(60.7) | 485(63.6) | 531(58.2) | 0.026 |
| Imageological examination |  |  |  |  |
| LVEDD (median[IQR]) | 58.0[52.0,65.0] | 56.0[51.0,62.0] | 60.0[53.0,68.0] | <0.001 |
| LVEF (median[IQR]) | 38.0[28.0,47.0] | 40.0[33.0,48.0] | 35.0[26.0,45.0] | <0.001 |
| LVEF<=40% (%) | 1006(60.1) | 402(52.7) | 604(66.2) | <0.001 |
| Global hypokinesis (%) | 422(25.2) | 110(14.4) | 312(34.2) | <0.001 |
| Hypokinesis (%) | 655(39.1) | 346(45.3) | 309(33.9) | <0.001 |
| Akinesis (%) | 998(59.6) | 537(70.4) | 461(50.5) | <0.001 |
| Apical LVT (%) | 1426(85.1) | 659(86.4) | 767(84.1) | 0.194 |
| Round LVT (%) | 922(55.0) | 387(50.7) | 535(68.7) | 0.001 |
| Mobile LVT (%) | 116(6.9) | 32(4.2) | 84(9.2) | <0.001 |
| Multiple LVT (%) | 168(10.0) | 43(5.6) | 125(13.7) | <0.001 |
| LVT largest diameter (median[IQR]) (mm) | 23.0[16.0,32.0] | 25.0[17.0,31.5] | 22.0[15.0,32.0] | 0.019 |
| LVT area (median[IQR]) (mm2) | 3.0[1.4,4.8] | 3.2[1.7,4.8] | 2.9[1.4,4.9] | 0.199 |

Abbreviations: ACEI=angiotensin-converting enzyme inhibitor. BPM=beats per minute. BMI=body mass index. CCB=calcium channel blocker. DBP=diastolic blood pressure. DM=diabetes mellitus. eGFR=estimated glomerular filtration rate. MI=myocardial infarction. CABG=coronary artery bypass grafting. PCI=percutaneous coronary intervention. SBP=systolic blood pressure. STEMI=ST-segment elevation myocardial infarction. NSTEMI=non-ST-segment elevation myocardial infarction. NVM=noncompaction of the ventricular myocardium. DAPT=dual antiplatelet therapy. VKA=vitamin-K antagonists. DOAC=direct oral anticoagulants. LVT=left ventricular thrombus. LVEDD=left ventricular end diastolic dimension. LVEF=left ventricular ejection fraction.**Supplementary Table 2 AIC and BIC of LCA cluster groups.**

| **Clusters** | **AIC** | **BIC** |
| --- | --- | --- |
| 2 | 17167.49 | 17332.14 |
| 3 | 16872.29 | 17121.76 |
| 4 | 16780.41 | 17114.69 |
| 5 | 16761.5 | 17180.6 |
| 6 | 16723.6 | 17227.52 |
| 7 | 16700.23 | 17288.98 |

Abbreviations: AIC, akaike information criterion; BIC, bayesian information criterion; LCA, latent class analysis.
